# Allelopathy and Identification of Volatile Components from the Roots and Aerial Parts of *Astragalus mongholicus* Bunge

**DOI:** 10.3390/plants13020317

**Published:** 2024-01-20

**Authors:** Xiu Wang, Yaqi Liu, Na Peng, Haitao Yu, Yu Ma, Mingxin Zhang, Yaoyao Wang, Yi Wang, Weiwei Gao

**Affiliations:** Institute of Medicinal Plant Development, Chinese Academy of Medical Sciences and Peking Union Medical College, Beijing 100193, China; wangxiu0510@163.com (X.W.); lyaqq0109@163.com (Y.L.); pengna@caas.cn (N.P.); thomasyu0126@163.com (H.Y.); 13681205244@163.com (Y.M.); z18437907377@126.com (M.Z.); wyy051402@163.com (Y.W.); 18854800636@163.com (Y.W.)

**Keywords:** *Astragalus membranaceus* var. *mongholicus*, volatile compounds, SPME-GC-MS, allelochemicals, autotoxicity, allelopathic activities, antifungal activity, antibacterial activity

## Abstract

The volatile compounds produced by plants play an important role in plant growth, plant communication, and resistance to biological and abiotic stresses. *Astragalus membranaceus* var. *mongholicus* (*AM*) is a perennial herbaceous plant (Leguminosae) that is widely cultivated in northwest China. The bioactive compounds in its root have shown various pharmacological activities. Root rot disease caused by *Fusarium* spp. often occurs in *AM* planting with increasing severity in continuous monoculture. It is currently still unclear what are the effects of the volatile compounds produced by fresh *AM* on itself, other crops cultivated on the same field after *AM*, pathogen, and rhizobia. In this study, we found that seed germination and seedling growth of *AM*, lettuce (*Lactuca sativa* L.), and wheat (*Triticum aestivum* L.) could be affected if they were in an enclosed space with fresh *AM* tissue. Additionally, 90 volatile compounds were identified by SPME-GC-MS from whole *AM* plant during the vegetative growth, 36 of which were specific to aerial parts of *AM* (stems and leaves, AMA), 17 to roots (AMR), and 37 were found in both AMA and AMR. To further identify the allelopathic effects of these volatile compounds, five compounds (1-hexanol, (*E*)-2-hexenal, (*E*,*E*)-2,4-decadienal, hexanal, and eugenol) with relatively high content in *AM* were tested on three receptor plants and two microorganisms. We found that (*E*,*E*)-2,4-decadienal and (*E*)-2-hexenal showed significant inhibitory effects on the growth of *AM* and lettuce. One-hexanol and hexanal suppressed the growth of wheat, while eugenol showed a similar effect on all three plant species. Moreover, the activities of these compounds were dose dependent. Notably, we discovered that (*E*)-2-hexenal and eugenol also inhibited the growth of the pathogen *Fusarium solani* by as high as 100%. Meanwhile, all five compounds tested suppressed the rhizobia *Sinorhizobium fredii*. In summary, this study furthered our understanding of the comprehensive allelopathic effects of the main volatile components of *AM*.

## 1. Introduction

*Astragalus mongholicus* is a perennial leguminous herb. The wild astragalus species are widely distributed in northern China, as well as in Kazakhstan, Mongolia, and the Far East of Russia [1,2,3,4]. As a traditional Chinese medicine, the dried roots of *AM* have been used to treat weakness, anemia, fever, allergies, chronic fatigue, loss of appetite, and uterine bleeding or prolapse [5]. Its extracts and active compounds have anti-inflammatory, antiviral, antioxidant, and immune-response activities [6]. One of the varieties, *Astragalus membranaceus* var. *mongholicus* (*AM*), is included in the current Chinese pharmacopeia. The demand for astragalus in the international market is also increasing [7]. China is the largest producer and consumer of astragalus in the world. At present, there are large areas of cultivation in northern China, and the commodities in the market mainly come from cultivation [8]. However, continuous monocultures generate a serious replant problem, and the yield and quality of astragalus have dropped dramatically [9]. The replant problem is a common trouble in the production of perennial medical plants. For astragalus, it generally takes more than two years for cultivation to reach the standard of medicine, while it takes about five years for wild astragalus. During continuous cropping, allelochemicals released by plants through volatilization, root exudation, rainwater leaching, and residue decomposition are continuously accumulated [10,11]. The plant volatiles deserve attention because of their different biological activities with plants [12], insects [13,14,15], microorganisms [16], etc. Allelopathy (especially autotoxicity allelopathy) and the accumulation of soil-borne pathogens, together with the imbalance of soil microbial communities caused by allelochemicals, were considered to be the basic factors of the replant problem [17,18,19]. Autotoxicity is best traced in Medicago sativa [20], which seriously affects the cultivation of alfalfa. The aqueous extract of dried astragalus had allelopathic activity on *Triticum aestivum* (wheat) [21]. Aqueous extract of the rhizosphere soil extract of cultivated *Astragalus hoantchy* had allelopathy in lettuce, wheat, corn, broad beans (*Vicia faba*), and flax (*Linum usitatissimum*) [22,23,24,25,26]. However, the composition of volatile components and the allelopathic potential of major compounds from two-year-old cultivated *AM* at the vegetative growth stage are still unclear.

The enrichment of rhizosphere pathogenic fungi caused by monocropping can lead to various plant root diseases [27]. For fungi, root rot is the most common disease of *AM*, with an incidence rate of more than 30% in China’s main production areas and 90% in severe cases [28]. The main pathogen is the *Fusarium* spp. [29,30]. The roots of leguminous plants are infected by rhizobia to form root nodules, then differentiate into bactericides in root nodules and fix nitrogen in the air as ammonia that can be absorbed and used by plants [31,32]. Astragalus is a leguminous plant that has a symbiosis with a variety of rhizobias, and the variety of rhizobias were isolated from astragalus in China [33]. Clarifying the interactions between the autotoxic factors of *AM* and pathogenic/beneficial microorganisms is also crucial.

Headspace solid-phase microextraction combined with gas chromatography–mass spectrometry (SPME-GC-MS) is widely used to identify volatile components of animals [34], plants [35], and fungi [36]. Compared with the traditional volatile oil extraction method combined with GC-MS analysis, it has the advantages of being simple, automatic, more sensitive, more accurate, and rapid [37,38], which helps to maintain the original flavor of volatile components. Therefore, in this study, we used SPME-GC-MS technology to identify the volatile components of *AM* in the field. We investigated the allelopathic activity of major volatile compounds on plants (*AM*, lettuce, and wheat), fungi (*Fusarium solani*), and bacteria (*Sinorhizobium fredii*). Finally, we provide a theoretical basis for rational rotation, new methods of disease control, and symbiotic bacteria rational application of *AM*.

## 2. Results

### 2.1. Allelopathy of Different Plant Parts

The allelopathic effects of the volatile compounds released by different parts of *AM* at 25 °C on three plants were explored. Placing plant seeds/seedlings and fresh plant samples in the same enclosed space verified that the volatiles emitted from the roots and aerial parts of *AM* had significant allelopathic effects on the seed germination and seeding growth of *AM*, lettuce, and wheat (Table 1). The results show that the volatile components released by the aerial parts of *AM* (stems and leaves, AMA) and roots (AMR) had autotoxicity on *AM*, and the allelopathic effect of the *AMA* treatment group was greater than that of AMR at the same concentration. AMA at 5 g/L had no effect on the germination rate (GR) or germination potential of *AM* seeds, but it significantly inhibited the growth of the radicle and shoots (SL), with inhibition rates of 70.15% and 46.59%, respectively. AMA in the 10 g/L treatment group significantly inhibited the seed germination and seedling growth of *AM* (*p* < 0.05), and the synthetic allelopathic index (SI) was −0.44. The allelopathic activity of the AMA treatment group had a dose-dependent effect, and the SI increased with increasing concentration. Compared with AMA, AMR showed an inhibitory effect on seed germination only when the concentration reached 40 g/L, and the inhibitory rates of GR and GP were 83.07% and 82.92%, respectively. The growth of the *AM* shoot was sensitive to the volatiles of AMR, which were significantly inhibited by 50.25% in the 5 g/L treatment group. The dose-dependent inhibitory effect of AMR-released volatiles on RL and SL increased, reaching 89.88% and 66.86% inhibition at 40 g/L concentration, respectively.

Volatile compounds from AMA caused a significant reduction in lettuce seed germination at doses ≥10 g/L. Lettuce seedlings exhibited greater vulnerability to the volatiles discharged by AMA. At the lowest dose in this experiment (5 g/L), the lengths of the radicle and shoot were 36.18% and 42.54% of the control; at the highest dose (40 g/L), the lengths of the radicle and shoot were only 8.03% and 11.79% of the control. The volatiles released from AMR had no effect on the germination rate of the lettuce seeds within the range of experimental settings; only at the highest dose (40 g/L) did it inhibit seed germination potential (26.41% inhibition rate). The lettuce seedling growth was inhibited in all treatment groups, with inhibition rates ranging from 19.97% to 85.05% for RL and 32.86% to 77.50% for SL.

When the dose was ≤20 g/L, the germination of the wheat seeds was not affected by the volatiles of AMA; at the 40 g/L dose, the GR and GP were 75.28% and 62.97% of the control, respectively. The wheat seedling growth was inhibited in all treatment groups, with inhibition rates ranging from 37.21% to 87.00% for RL and 6.86% to 77.79% for SL. Volatiles from AMR had an impact on wheat seed germination only at high concentrations (40 g/L), resulting in 42.69% and 41.38% inhibition for GR and GP, respectively. When the dose was ≥20 g/L, the growth of radicles and shoots was inhibited.

### 2.2. Analysis of Volatile Constituents in the Aerial Parts and Roots of Fresh AM

A total of 90 volatile components were identified (Table S1). These included 23 alcohols, 19 lipids, 16 aldehydes, 2 phenolic compounds, 7 acids, 11 ketones, 3 alkenes, 7 heterocycles, and 2 other compounds. Thirty-seven compounds were the same components in the two groups. Alcohols and aldehydes accounted for a large proportion of volatile components in the aerial parts of the *AM* (AMA) and roots (AMR) groups.

Seventy-three volatile components were identified in AMA, including 21 alcohols (19.57%), 14 aldehydes (18.36%), 17 lipids (16.50%), 9 ketones (11.81%), 5 heterocycles (4.18%), 3 acids (3.658%), 2 phenolic compounds (0.68%), 1 alkenes (0.21%), and 1 other compounds (0.21%), accounting for 75.24% of the total volatile components (Table S1). Among them, the 10 compounds with the highest relative content were hexanal, benzaldehyde, (*Z*)-3-Hexen-1-ol, 1-Penten-3-one, benzyl alcohol, Methyl Alcohol, 1-Penten-3-ol, 4-(2,6,6-trimethyl-1-cyclohexen-1-yl)-3-Buten-2-one, 1-Octen-3-one, and (*Z*)-Butanoic acid-3-hexenyl ester (Table 1). The details of other compounds are shown in Table S1.

Fifty-four volatile components were identified in AMR, including 10 alcohols (25.21%), 14 aldehydes (19.77%), 6 acids (8.21%), 5 heterocycles (7.46%), 8 ketones (4.99%), 6 lipids (3.97%), 2 phenolic compounds (0.40%), 2 alkenes (0.47%), and 1 other compounds (0.17%), accounting for 70.65% of the total volatile components. The details of other compounds are displayed in Table S1. Among them, the compounds with the highest relative content (top 10) were 1-hexanol, 2-octanol, hexanoic acid, hexanal, benzaldehyde, 1-octen-3-ol, 2-pentyl-Furan, (*E*)-2-hexenal, 2-methoxy-3-(1-methylpropyl)-Pyrazine, and 2-ethyl-Furan (Table 2).

### 2.3. Allelopathy of Main Volatile Compounds from AM

One-hexanol and hexanal, as the most abundant compounds in AMR and AMA, respectively, were selected for activity verification. Moreover, (*E*)-2-hexenal was reported as the most abundant aldehyde (8.67%) in fresh astragalus, followed by hexanal (5.4%) [39]. Moreover, according to recent articles, (*E*,*E*)-2,4-decadienal and eugenol have a variety of biological activities, such as antioxidant and antibacterial activities [40,41]. Therefore, (*E*)-2-hexenal, (*E*,*E*)-2,4-decadienal, and eugenol were also chosen.

#### 2.3.1. Allelopathic Activities of the Compounds on AM

**Figure 1:**
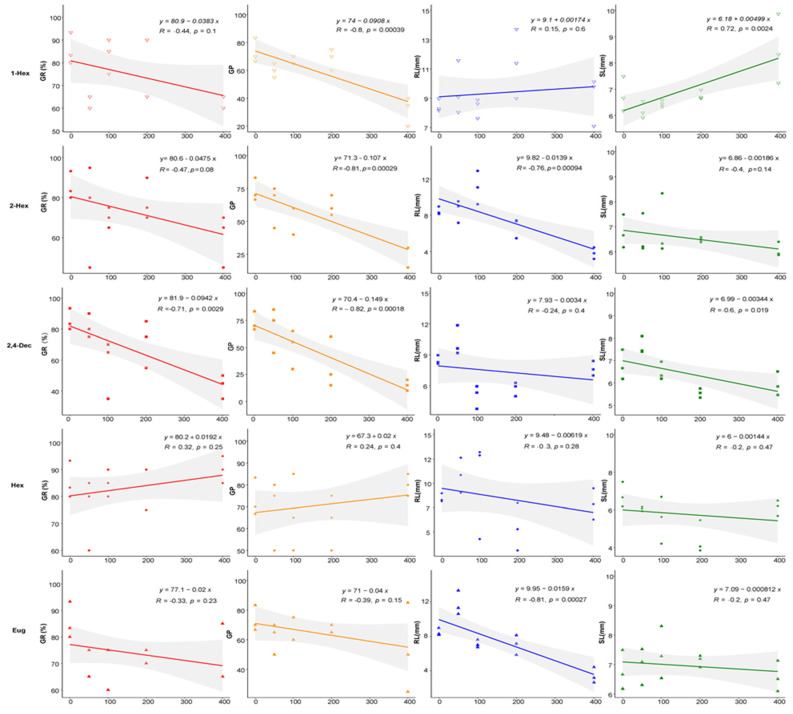
Allelopathic activities of compounds on seeds and seedlings of *AM.* Notes: 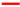 GR, germination rate; 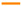 GP, germination potential; 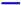 RL, radicle length; 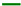 SL, shoot length; 1-Hex: 1-hexanol; 2-Hex: (*E*)-2-hexenal; 2,4-Dec: (*E*,*E*)-2,4-decadienal; Hex: hexanal; Eug: eugenol.

The effects of five compounds on the seed germination and seedling growth of *AM* were different (Figure 1). 1-hexanol significantly inhibited the germination of seeds, and the dose effect was very significant: the seed germination rate and germination potential in the 400 mg/L treatment group were 72.09% and 43.19% of the control, respectively. It had no significant effect on the radicle, but the shoot length treated with 400 mg/L was significantly greater than that of the control. (*E*)-2-hexenal had no significant effect on the seed germination rate, but it reduced the seed germination potential: in the 400 mg/L treatment, the inhibition of the seed GP reached 66%. It significantly inhibited the radicle growth but not the shoot in the 400 mg/L treatment group, and the SI was—0.40. (*E*,*E*)-2,4-decadienal significantly inhibited the seed germination and inhibited the radicle growth and shoot length at high doses; the SI of the 400 mg/L group was—0.38. Hexanal had no obvious effect on seed germination or seedling growth. Eugenol had no effect on the seed germination or shoot length of seedlings. The radicle length of seedlings was significantly lower in the 400 mg/L treatment group than in the control group (0.41 times that of the control). All statistical analyses are shown in Table S2.

#### 2.3.2. Allelopathic Activities of the Compounds on Lettuce

**Figure 2:**
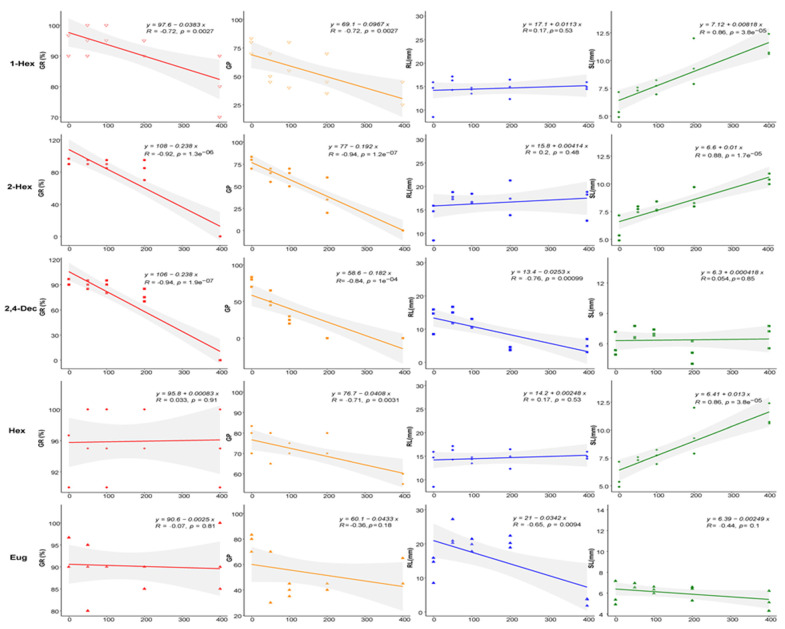
Allelopathic activities of compounds on seeds and seedlings of lettuce. Notes: 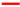 GR, germination rate; 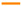 GP, germination potential; 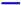 RL, radicle length; 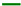 SL, shoot length; 1-Hex: 1-hexanol; 2-Hex: (*E*)-2-hexenal; 2,4-Dec: (*E*,*E*)-2,4-decadienal; Hex: hexanal; Eug: eugenol.

As shown in Figure 2, 1-hexanol significantly inhibited the germination of lettuce seeds at the concentration of 400 mg/L; the inhibition rates of the germination rate and germination potential were 15.30% and 59.28%, respectively. It promoted the growth of seedlings, and the dose effect was significant. A low concentration (≤200 mg/L) of (*E*)-2-hexenal had no effect on lettuce seed germination, and the inhibition rate reached 100% when the concentration was 400 mg/L. It promoted the growth of the shoots, and the promotion effect was positively correlated with the concentration. (*E*,*E*)-2,4-decadienal significantly inhibited seed germination, and the GR of the 400 mg/L treatment group was zero (SI of −0.40). It had no significant effect on the shoot length or seeding fresh weight. The radicle length of the seedlings in the 200 mg/L and 400 mg/L treatment groups was significantly smaller than that in the control group (0.31 and 0.38 times that of the control). Hexanal had no effect on lettuce seed germination or the radicle growth of seedlings, but it significantly promoted shoot growth, and the dose effect was obvious (1.94 times that of the control in the 400 mg/L group). Eugenol did not affect the seed germination rate, but it reduced the seed germination potential. It showed a trend of low-concentration promoting and high-concentration inhibiting radicle growth: the radicle length was the largest in the 50 mg/L treatment group (1.75 times that of the control) and the smallest in the 400 mg/L treatment group (0.24 times that of the control). All statistical analyses are shown in Table S3.

#### 2.3.3. Allelopathic Activities of the Compounds on Wheat

**Figure 3:**
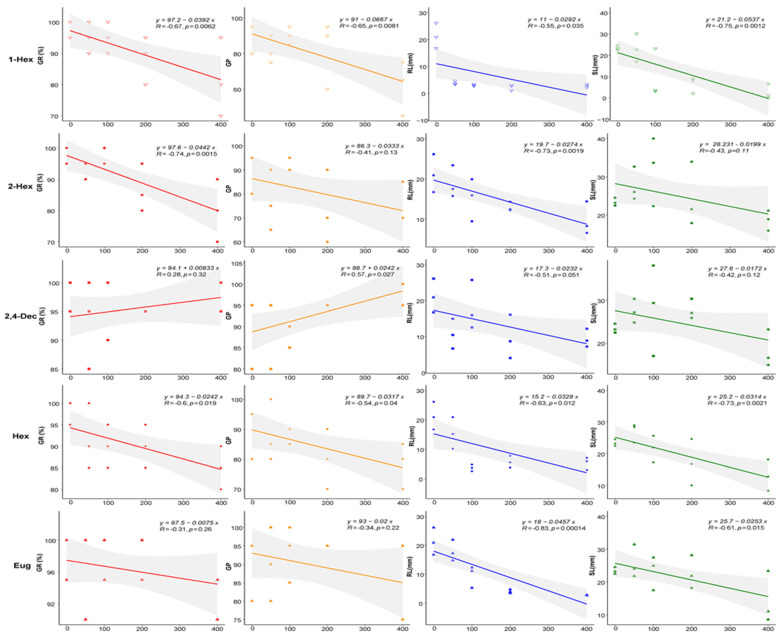
Allelopathic activities of compounds on seeds and seedlings of wheat. Notes: Notes: 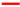 GR, germination rate; 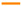 GP, germination potential; 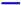 RL, radicle length; 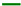 SL, shoot length; 1-Hex: 1-hexanol; 2-Hex: (*E*)-2-hexenal; 2,4-Dec: (*E*,*E*)-2,4-decadienal; Hex: hexanal; Eug: eugenol.

As shown in Figure 3, 1-hexanol had no effect on seed germination but had an inhibitory effect on the growth of seedlings, and the dose effect was obvious: the SI reached —0.56 at 400 mg/L. (*E*)-2-hexenal significantly reduced the germination rate of the wheat seeds but had no significant effect on the seed germination potential at 400 mg/L. The growth of the seedling shoots was generally promoted by a low concentration and inhibited by a high concentration, and the shoot length was the highest at 100 mg/L (1.37 times that of the control). (*E*,*E*)-2,4-decadienal had no effect on wheat seed germination or seedling shoot, but when the concentration reached 200 mg/L, it significantly inhibited the growth of the seedling radicle, with an inhibition rate of 54.56%. Hexanal did not affect seed germination, but it significantly inhibited radicle growth, and the inhibitory effect increased with increasing concentration. Eugenol had no obvious effect on the seed germination but showed a trend of low-concentration promoting and high-concentration inhibiting the growth of seedling shoots. At a concentration of 400 mg/L, it inhibited the seed germination and seedling growth the most significantly (SI = −0.35). All statistical analyses are shown in Table S4.

### 2.4. Antifungal Activity of the Compounds on F. solani

**Figure 4:**
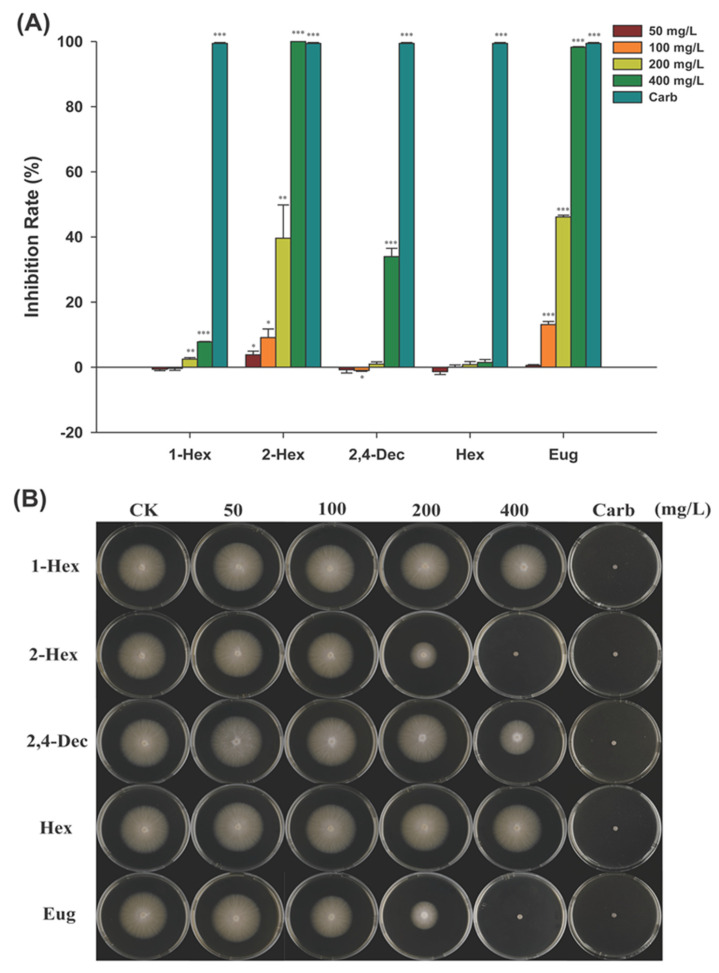
Antifungal activity on *F. solani* of compounds. (**A**) Inhibition rate of monomer compounds on *F. solani*; (**B**) Typical photos of the inhibition of *F. solani* growth by monomer compounds. The data collection was carried out on the 5th day. 1-Hex: 1-hexanol; 2-Hex: (*E*)-2-hexenal; 2,4-Dec: (*E*,*E*)-2,4-decadienal; Hex: hexanal; Eug: eugenol; Carb, carbendazol; Student’s *t*-test, * *p* < 0.05, ** *p* < 0.01, *** *p* < 0.001.

As shown in Figure 4, five compounds all showed the phenomenon of high concentrations inhibiting the growth of fungi, but the inhibition intensity of the different compounds was dissimilar. The inhibitory activity of 1-hexanol against *F. solani* was low, and the inhibition rate was only 7.79% when the concentration reached 400 mg/L. (*E*)-2-hexenal showed an inhibition rate of 3.83% at a concentration of 50 mg/L and of 100% at a concentration of 400 mg/L. (*E*,*E*)-2,4-decadienal with a concentration lower than 100 mg/L promoted the growth of *F. solani*; when the concentration reached 200 mg/L, it began to show inhibition, and the inhibition rate reached 33.96% at 400 mg/L. Although hexanal had a trend of low promotion and high inhibition, its overall effect was very low. The inhibition rate of eugenol reached 13.12% at 100 mg/L and 100% at 400 mg/L.

### 2.5. Antibacterial Activity of the Compounds on S. fredii

**Figure 5:**
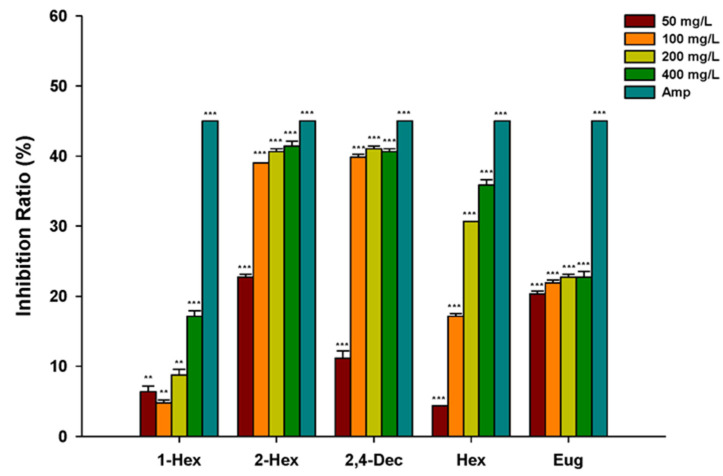
Inhibition rate of compounds on *S. fredii*. 1-Hex: 1-hexanol; 2-Hex: (*E*)-2-hexenal; 2,4-Dec: (*E*,*E*)-2,4-decadienal; Hex: hexanal; Eug: eugenol; Amp, ampicillin; Student’s *t*-test, ** *p* < 0.01, *** *p* < 0.001.

As shown in Figure 5, five compounds showed an inhibition of *S. fredii* growth, but the inhibition intensity was not equal. The inhibitory activity of 1-hexanol against *S. fredii* was low, and the inhibition rate was only 17.13% when the concentration reached 400 mg/L. The inhibition rates of (*E*)-2-hexenal and (*E*,*E*)-2,4-decadienal reached 39.04% and 39.84% at a concentration of 100 mg/L, respectively. When the concentration reached 100 mg/L, increasing the concentration of the compound had no significant effect on the inhibition. Hexanal showed significant inhibition at a concentration of 100 mg/L (inhibition rate of 17.13%), and the inhibition rate increased with the increase in the compound dose (35.85% at 400 mg/L). The inhibition rate of eugenol reached 20.32% at a concentration of 50 mg/L, and the inhibition rate did not change significantly with the increase in compound concentration.

## 3. Discussion

Autotoxicity of plant volatiles is a relatively common physiological phenomenon. It has been reported in several plant species, such as *Artemisia herba-alba* [4], *Amomum villosum Lour* [42], and *Hordeum vulgare* L. [43]. In this study, the volatile compounds have autointoxication on *AM*, and the dose effect was significant. At the same dose, it was found that the volatiles of AMA samples had stronger allelopathic effects on the three receptor plants than AMR. This may be due to the higher abundance of volatiles released from AMA compared with those from AMR. GR represents the germination ability, and GP represents the germination capacity and uniformity [44]. The effects of volatiles on GR and GP in our study followed similar trends; meanwhile, GP demonstrated greater sensitivity to changes in compound dosage. This phenomenon was also observed in studies involving the volatiles or extracts of other plants, such as Canada goldenrod (*Solidago canadensis* L.) [45] and horseweed (*Conyza canadensis* L.) [46].

In this study, the SPME method was used to collect volatile compounds, and 90 volatile components were identified from two-year-old fresh *AM* samples, 48 of which were reported (fresh and dry samples of *AM*), and 42 were not reported as volatile components of *AM*. Xu et al. used simultaneous distillation extraction (SDE) to extract essential oil from fresh *AM*, obtaining 43 volatile components [39].This may be due to essential oil loss and distortion caused by the higher extraction temperature of the SDE method. Alcohols had the most species and highest content of volatile compounds in our study, while this was the case for aldehydes in volatile oil from dried astragalus in the literature [39,47,48]. During sample drying, the loss of alcohols and the increase in aldehydes are caused by the Maillard reaction, lipid oxidation and degradation, the interaction between oxygenated lipids and amino acids or proteins, and long-chain- compound degradation [49,50,51]. The plants used in this work were fresh AMA in the vegetative growth stage, while those used in other studies were collected in the harvest season; most of the reported volatile components of astragalus were identified in dried samples, and few of them were from fresh *AM*. The plant origins, processing methods, volatile oil extraction conditions, detection and identification methods, and other conditions of experimental samples all cause differences in volatile components and contents [39,52,53,54]. All the above factors may be related to the large differences between the volatile oil components determined in this study and those in the literature.

In this study, five compounds were selected for bioassay experiments: 1-hexanol, hexanal, (*E*)-2-hexenal, (*E*,*E*)-2,4-decadienal, and eugenol. 1-hexanol accounted for 8.23% of the volatile components in the AMR, representing the most abundant compound. As the major volatile component of the soybean odor of Radix Astragali [55], hexanal was the most abundant compound among the volatile components of AMA, with a relative content of 7.05%. (*E*)-2-hexenal accounted for 2.39% of volatile components in our sample, and Xu et al. reported that the relative percentage content of (*E*)-2-hexenal is 8.67%, which is the most abundant aldehyde in fresh astragalus, followed by hexanal (5.4%) [39]. According to recent articles, (*E*,*E*)- 2,4-decadienal and eugenol have a variety of biological activities, such as antioxidant and antibacterial activities [40,41]. (*E*,*E*)-2,4-decadienal and eugenol were not identified in our study, which may be due to the different sources and processing methods of the test samples. There were reports that the content of volatile components of (*E*,*E*)-2,4-decadienal in fresh astragalus is 2.12% [39], and eugenol accounts for 1.09% of the volatile components in dried astragalus [47]. Therefore, we also selected these two compounds for activity verification.

Five compounds were observed to have allelopathic effects on *AM* and the other two receptor plants: lettuce (dicotyledon) and wheat (monocotyledon). (*E*,*E*)-2,4-decadienal is a lipid peroxidation product that has been detected in a variety of plant volatile oils [39,56]. This study found that (*E*,*E*)-2,4-decadienal has autotoxicity to *AM* and could inhibit seed germination and seedling growth. At the same time, it has obvious allelopathy on lettuce and wheat. (*E*)-2-hexenal has been reported to affect the morphogenesis of plant roots by inhibiting growth and development [57,58]. This study found that it inhibited the growth of *AMA* and wheat seedlings and inhibited the biomass accumulation of lettuce seedlings, which is consistent with the latest literature reports. In this work, eugenol promoted the growth of lettuce at a low concentration (≤ 200 mg/L) and inhibited growth at a high concentration (≥400 mg/L) on lettuce. It has a significant inhibitory effect on wheat growth, which is similar to what is found in the literature [59]. Although it was not identified in this work, its role is worth considering as it has been reported in other studies of *AM* volatiles. Hexanal is a compound commonly found in plant essential oil. This study found that it could significantly promote the biomass accumulation of AMA in a low dose and inhibit the growth of wheat but had no effect on the growth of *L. sativa*. 1-hexanol is a common alcohol compound in plant essential oil; it has been reported to be related to caterpillar attraction in *Spodoptera littoralis*, *Bemisia tabaci* (Gennadius) (Hemiptera: Aleyrodidae), etc. [60,61], but its effects on plants are limited. It inhibited the germination of AMA and lettuce seeds but promoted their seedling growth. Interestingly, 1-hexanol has no effect on the germination of wheat seeds, but it significantly inhibits the growth of wheat seedlings. The physiological difference between dicotyledons and monocotyledons may be the reason for this phenomenon.

Heras-Mozos et al. reported that antifungal packaging based on (*E*)-2-hexenal could extend the shelf life of strawberries [62]. It could also completely inhibit *F. graminearum*, *Aspergillus flavus*, and *A. niger,* potentially controlling pests at the same time [63,64]. In this study, (*E*)-2-hexenal at a concentration of 200 mg/L was effective against *F. solani*. These results also showed an obvious inhibition effect in an increased concentration. We speculated that (*E*)-2-hexenal may play an important role in the process of resistance to pathogenic fungi (*F. solani*) during the growth of AMA. Eugenol has antifungal activity against *Penicillium*, *Aspergillus*, and *Fusarium* [65] and activity against a wide range of gram-negative and gram-positive bacteria [16]. All five volatile oil monomers have significant inhibitory activity on *S. fredii.* Leguminous plants have an almost unique symbiotic ability with nitrogen-fixing rhizobia in the soil [66]. Researchers isolated a variety of rhizobia from astragalus in China [33]. However, in agriculture, we found that compared with legumes such as soybeans, there are few root nodules in *A. mongholicus*. This suggested that the inhibition of volatile components on rhizobia may be one of the reasons why *A. mongholicus* has fewer nodulates.

## 4. Materials and Methods

### 4.1. Samples

The plant used in this study is a two-year-old *AM,* which is in the vegetative growth stage. The seedlings come from the planting base of *AM* in Hohhot, China. It was identified as *Astragalus mongholicus* Bunge (syn. *A.membranaceus* (Fisch.) Bunge var. *mongholicus* (Bunge) P. K. Hsiao) by Professor Bengang Zhang from the Institute of Medicinal Plants, Chinese Academy of Medical Sciences, Beijing, China. Three kilograms of aerial parts/roots of fresh astragalus were collected randomly from the field. The samples were cut into 1–2 cm pieces and thoroughly mixed. Subsequently, 1 kg of the samples was stored at −80 °C for SPME-GC-MS experiments, while the remaining samples were used for plant allelopathy experiments.

### 4.2. SPME-GC-MS

In the SPME cycle of the PAL railway system, a measure of 1000 ± 5 mg of powder sample was put into a 20 mL headspace bottle, and 10 μL 2-octanol (10 mg/L in double-distilled water, ddH_2_O) was added as internal standard. It was then pre-heated at 60 °C for 15 min and incubated for 30 min to make the volatile components gather above the headspace bottle. The solid phase extraction needle was inserted into the headspace bottle to absorb all volatile components and then injected into the GC-MS instrument for identification. GC-MS analysis was performed using an Agilent 7890 gas chromatograph system coupled with a 5977B mass spectrometer. The system used a DB-Wax, in-column splitless injection mode. Helium was used as the carrier gas, the front inlet purge flow was 3 mL·min^−1^, and the gas flow rate through the column was 1 mL·min^−1^. The initial temperature was kept at 40 °C for 4 min, then raised to 245 °C at a rate of 5 °C·min^−1^ and maintained for 5 min. The injection, transfer line, ion source, and quad temperatures were 250, 250, 230, and 150 °C, respectively. The energy was −70 eV in electron-impact mode. The mass spectrometry data were acquired in scan mode, with a range of 20–400 m/z and a solvent delay of 0 min.

GC-MS data analysis was conducted as follows: Chroma TOF 4.3X software of LECO Corporation and NIST database were used for raw peak exacting, data baselines filtering and calibration of the baseline, peak alignment, deconvolution analysis, peak identification, and integration of the peak area. The peak area normalization method was used for the relative quantitative analysis of volatile components.

### 4.3. Allelopathy of Volatile Compounds Released by Plants

In accordance with the published literature [42], the prepared plant samples were placed in an airtight plastic container (volume 1.2 L) at the following concentrations (plant mass/container volume): 0 g/L, 5 g/L, 10 g/L, 20 g/L, and 40 g/L. Three replicates were set up for each treatment. Sterile filter paper was placed in a 10 cm square petri dish (Beijing Jiang chen wen xuan Biotechnology Co., Ltd., Beijing, China), and 2 mL of sterile water was added to moisten the filter paper.

(1) Seed germination experiment: *AM*, lettuce, and wheat seeds of uniform size and full shape were selected and washed with distilled water and set aside. The seeds were sterilized using 75% alcohol for a minute and washed three times with ddH_2_O. Following this, they were treated with 1.0% NaClO for two minutes (wheat for 10 min), washed three times with ddH_2_O, and finally placed on a sterile operating platform to air-dry. The treated seeds were placed in square petri dishes with 30 seeds per dish. Petri dishes were placed in containers without lids. The containers were sealed using a sealing film and then placed in a climatic chamber (25 ± 1 °C) for incubation. Germination was standardized by penetrating the radicle or germ into the seed coat by 1–2 mm. The number of germinated seeds was recorded daily until no new seeds germinated for three consecutive days. The number of seeds germinated per day was recorded, and germination rate (GR), germination potential and chemotaxis index were calculated.

(2) Seedling growth experiment: Healthy and similar pre-germinated seeds were selected for the experiment. Ten germinated seeds were placed evenly in each petri dish. Petri dishes were placed diagonally in the containers without lids. The containers were sealed with a sealing film. After five days of incubation in an artificial climate chamber (25 ± 1 °C, L:D = 16:8), SL (shoot length) and RL (radicle length) were recorded (six days for lettuce). The combined SI (synthetical allelopathic index) was calculated. Calculation methods of Wang et al. [67] were quoted:

$$\mathrm{GR}=\frac{number\;of\;germinated\;seeds}{number\;of\;tested\;seeds}\times 100\%$$

$$\mathrm{GP}=\frac{\mathrm{number}\mathrm{of}\mathrm{seeds}\mathrm{germinated}\mathrm{at}\mathrm{the}\mathrm{peak}\mathrm{of}\mathrm{germination}}{\mathrm{number}\mathrm{of}\mathrm{tested}\mathrm{seeds}}\times 100\%$$

$$\mathrm{RI}=\frac{\mathrm{treatment}\mathrm{value}}{\mathrm{control}\mathrm{value}}\;–1$$

SI = (RIGR + RIGP + RIRL + RISL)/4

### 4.4. Allelopathy of Main Volatile Compounds

According to the identification results of volatile oil obtained in this study and reports in the literature [39,40,41,47], five compounds with a relatively high percentage of volatile oil components were selected for bioassay experiments: 1-hexanol (CAS: 111-27-3, Aladdin, Shanghai Aladdin Biochemical Technology Co., Ltd., Shanghai, China), (*E*)-2-hexenal (CAS: 6728-26-3, Aladdin), (*E*,*E*)-2,4-decadienal (CAS: 25152-84-5, AladdinShanghai Aladdin Biochemical Technology Co., Ltd., Shanghai, China), hexanal (CAS: 66-25-1, Macklin, Shanghai Macklin Biochemical Technology Co., Ltd., Shanghai, China), and eugenol (CAS: 97-53-0, Aladdin, Shanghai Aladdin Biochemical Technology Co., Ltd., Shanghai, China). The compounds were dissolved to a concentration of 40,000 mg/L using acetone and were used as a mother liquor. The mother liquor was then diluted with ddH_2_O, with final concentrations of 400, 200, 100, and 50 mg/L. The ddH_2_O with 1% acetone was used as a control. The methods of seed germination and the seeding growth experiment were carried out according to the methods by Han et al. [54], with some adjustments made.

(1) Seed germination experiment: The sterile filter paper was placed in a 10 cm square culture dish, and 2 mL of compound diluent of a specific concentration was added to soak the filter paper; three replicates (dishes) were set for each treatment. Twenty treated seeds were placed in each dish, and the petri dishes were sealed using a sealing film. Refer to Section 4.4 for seed treatment, incubation conditions, and statistical scheme.
(2) Seedling growth experiment: Healthy and similar pre-germinated seeds were selected for the experiment. A filter paper was placed in a 10 cm square culture dish, and 1 mL of volatile compound solution of the corresponding concentration was added. A measure of 1 mL of the corresponding compound solution was added on the fourth day. The petri dish was sealed with a sealing membrane after each operation. Refer to Section 4.4 for incubation conditions and statistical scheme.

### 4.5. Allelopathic Activity on Pathogen

The growth rate method was used to determine the antifungal activity [68,69,70]. The pathogen used in this research was *Fusarium solani* (*F. solani*), sourced from the Molecular Ecology Laboratory of the Institute of Medicinal Plants, Chinese Academy of Medical Sciences, Beijing, China. The strain was isolated from the root samples of *AM* infected with root rot and identified by Xiu Wang. On a clean bench, PDA with final concentrations of five compounds of 400, 200, 100, and 50 mg/L, respectively, were prepared. The Petri dishes used were 9 cm in diameter. Four-millimeter colony discs were placed in the center of the PDA dish containing volatile components of different concentrations, with one placed in each dish. The experiment was performed three times. PDA with 1% (*v*/*v*) acetone was used as a blank control (compound concentration of 0 mg/mL). PDA with 800 mg/L carbendazol was used as a positive control. After sealing them with sealing film, they were incubated for five days in the dark at 25 °C. The colony growth diameter was measured using a vernier caliper, and the mycelial inhibition rate was calculated [71].

$$\mathrm{Inhibition}\;\mathrm{rate}\;(\%)=\frac{colony\;diameter\;of\;control-colony\;diameter\;of\;treatment}{colony\;diameter\;of\;control-4\mathrm{mm}}\;\times 100\%$$

### 4.6. Allelopathic Activity on Rhizobia

The experimental method of bacterial inhibitory activity was adjusted according to the literature [70,72]. The strain used in the experiment was *Sinorhizobium fredii* (BeNa Culture Collection, BNCC), which was cultured in YM [73] at 25 °C and 130 rpm for 24 h, then prepared into a concentration of 1 × 10^7^ CFU/mL bacterial suspension. On an ultra-clean bench, YM with a final concentration of 400, 200, 100, and 50 mg/L, respectively, were prepared. YM of 1% acetone was taken as blank control; YM containing 100 mg/L ampicillin was used as the positive control. A measure of 1 mL of the prepared culture solution was taken for each treatment, and 10 μL of bacterial suspension was added, then incubated with constant temperature oscillation (25 ± 1 °C, 130 r/min) for 24 h; YM was taken as a control, and the OD_600_ value of each treatment was measured. There were three replicates (dishes) for each treatment. The inhibition rate of each treatment was calculated [74].

$$\mathrm{Inhibition}\;\mathrm{rate}\;(\%)=\frac{OD600\;of\;control-OD600\;of\;treatment}{OD600\;of\;control}\;\times 100\%$$

### 4.7. Statistical Analysis

All data were analyzed using SPSS 21 (IBM, Armonk, NY, USA) and R version 4.3.2 (R Core Team, Vienna, Austria). The significant difference between the treatment group and the control group was calculated by one-way ANOVA. Statistical significance was accepted at *p* < 0.05. SigmaPlot 10.0 (SYSTAT, San Jose, CA, USA) software was used to draw graphs.

## 5. Conclusions

We found that 73 volatile compounds were released from the stem and leaves of fresh *A. mongholicus*, which are more than the 54 released from the roots. The volatiles inhibited the seed germination and seedling growth of astragalus, lettuce, and wheat. The allelopathic activity of volatiles released from stems and leaves was stronger than that from roots. Five volatile compounds, namely 1-hexanol, (E)-2-hexenal, (*E*,*E*)-2,4-decadienal, hexanal, and eugenol, had an inhibitory effect on *F. solani* and *S. fredii*. Moreover, these five compounds had different allelopathic activity on the three receptor plants. These results suggested that the volatiles released by *A. mongholicus* may play a role in the growth of crops and the reproduction of beneficial/harmful microorganisms. Our study will help us to understand the comprehensive effects of volatiles released during the *A. mongholicus* growth process.

## Figures and Tables

**Table 1:** Allelopathic activities of volatiles released by AMA and AMR on three receptor plants.

Treatment	Plants	Concentration (g/L)	Seeds		Seedings		SI
			GR (%)	GP	RL (mm)	SL (mm)	
AMA	*AM*	0	91.11 ± 1.11 a	81.11 ± 1.11 a	33.87 ± 6.59 a	14.08 ± 3.03 a	0.00
		5	85.50 ± 2.22 a	81.67 ± 0.96 a	10.11 ± 0.63 b	7.52 ± 0.25 b	−0.31
		10	60.00 ± 1.92 b	55.00 ± 4.81 b	8.01 ± 0.54 b	6.61 ± 0.12 b	−0.44
		20	52.22 ± 10.60 b	38.33 ± 2.89 c	5.35 ± 0.34 b	4.31 ± 0.26 b	−0.60
		40	14.44 ± 4.84 c	14.44 ± 4.84 d	3.42 ± 0.06 b	3.25 ± 0.58 b	−0.84
	Lettuce	0	91.11 ± 1.11 a	88.33 ± 0.96 a	52.04 ± 3.11 a	18.57 ± 0.21 a	0.00
		5	92.22 ± 1.11 a	86.67 ± 3.85 a	18.83 ± 1.42 b	7.90 ± 0.33 b	−0.30
		10	80.00 ± 1.92 b	80.00 ± 1.92 ab	15.2 ± 1.92 bc	7.17 ± 0.41 bc	−0.35
		20	73.33 ± 0.00 c	73.33 ± 0.00 b	9.23 ± 2.34 cd	4.82 ± 1.04 c	−0.47
		40	0.00 ± 0.00 d	0.00 ± 0.00 c	4.18 ± 0.09 d	2.19 ± 0.11 d	−0.95
	wheat	0	98.89 ± 1.11 a	90.00 ± 1.93 ab	87.98 ± 1.59 a	44.57 ± 2.60 a	0.00
		5	97.78 ± 2.22 a	96.67 ± 1.93 a	55.24 ± 5.83 b	38.48 ± 3.31 ab	−0.13
		10	95.56 ± 2.94 a	93.33 ± 1.93 a	42.54 ± 6.06 b	31.84 ± 2.28 bc	−0.21
		20	92.22 ± 1.11 a	76.67 ± 0.00 b	22.00 ± 2.06 c	23.78 ± 3.03 c	−0.36
		40	74.44 ± 4.84 b	56.67 ± 5.77 c	11.44 ± 1.44 c	9.90 ± 1.28 d	−0.54
AMR	*AM*	0	91.11 ± 1.11 a	81.11 ± 1.11 a	33.88 ± 6.59 a	14.09 ± 3.03 a	0.00
		5	85.56 ± 2.94 a	71.11 ± 4.44 a	22.37 ± 2.53 ab	7.01 ± 0.27 b	−0.27
		10	84.45 ± 4.01 a	74.44 ± 5.88 a	10.95 ± 0.98 bc	7.57 ± 0.27 b	−0.35
		20	80.00 ± 0.00 a	63.33 ± 5.09 a	6.99 ± 0.44 c	6.31 ± 0.46 b	−0.43
		40	15.56 ± 2.94 b	15.56 ± 2.94 b	3.43 ± 0.08 c	4.67 ± 0.11 b	−0.79
	Lettuce	0	91.11 ± 1.11 a	88.33 ± 0.96 a	52.04 ± 3.11 a	19.02 ± 0.34 a	0.00
		5	84.44 ± 2.94 a	83.33 ± 3.85 a	41.65 ± 0.37 b	12.77 ± 0.86 b	−0.16
		10	94.44 ± 1.11 a	90.00 ± 0.00 a	34.14 ± 0.79 b	11.20 ± 1.14 bc	−0.17
		20	91.11 ± 4.84 a	78.33 ± 0.96 ab	23.29 ± 2.24 c	7.48 ± 0.83 cd	−0.29
		40	81.11 ± 9.69 a	65.00 ± 6.74 b	7.78 ± 2.04 d	4.28 ± 1.14 d	−0.46
	wheat	0	98.89 ± 1.11 a	96.67 ± 0.00 a	82.29 ± 2.70 a	51.33 ± 2.55 a	0.00
		5	100.00 ± 0.00 a	97.78 ± 2.22 a	79.59 ± 0.90 a	44.44 ± 0.75 ab	−0.02
		10	96.67 ± 1.93 a	93.33 ± 3.33 ab	74.90 ± 3.04 a	45.41 ± 1.82 ab	−0.07
		20	95.56 ± 2.94 a	86.67 ± 5.09 ab	53.03 ± 4.40 b	41.05 ± 1.33 b	−0.18
		40	56.67 ± 5.77 b	56.67 ± 5.77 b	30.47 ± 0.49 c	23.99 ± 2.70 c	−0.49

Notes: GR, germination rate; GP, germination potential; RL, radicle length; SL, shoot length; SI, synthetical allelopathic index; Different lowercase letters indicate significant difference at *p* < 0.05 level.

**Table 2:** Top 10 compounds of volatile components in aerial parts and roots of *AM*.

NO.	Aerial Parts (AMA)			Roots (AMR)		
	Name	RI^a^/RI^b^	RC %	Name	RI^a^/RI^b^	RC %
1	Hexanal	1074/1083	7.05	1-Hexanol	1344/1355	8.23
2	Benzaldehyde	1497/1520	6.24	2-Octanol	1411/1411	6.79
3	(*Z*)-3-Hexen-1-ol, acetate	1291/1316	5.59	Hexanoic acid	1835/1846	6.78
4	1-Penten-3-one	1011/1019	3.66	Hexanal	1074/1083	5.68
5	Benzyl alcohol	1869/1870	2.86	Benzaldehyde	1497/1520	5.33
6	Methyl Alcohol	931/903	2.62	1-Octen-3-ol	1438/1450	4.52
7	1-Penten-3-ol	1159/1158	2.34	2-pentyl-Furan	1212/1232	2.94
8	3-Buten-2-one, 4-(2,6,6-trimethyl-1-cyclohexen-1-yl)-	1929/1967	2.32	(*E*)-2-Hexenal	1185/1216	2.39
9	1-Octen-3-one	1282/1301	2.28	Pyrazine, 2-methoxy-3-(1-methylpropyl)-	1484/1502	1.8
10	(*Z*)-Butanoic acid, 3-hexenyl ester	1443/1455	2.24	2-ethyl-Furan	960/951	1.63

Notes: RT, retention time; RC, relative content; RI: Retention Index; a, calculated RI; b, RI in NIST or literature.

## Data Availability

Data is contained within the article and Supplementary Material.

## Supplementary Materials

The following supporting information can be downloaded at: https://www.mdpi.com/article/10.3390/plants13020317/s1, Table S1. Identity of volatile compounds in *Astragalus mongholicus* (*AM*). Table S2. Allelopathic activities of compounds on seeds and seedings of *AM.* Table S3. Allelopathic activities of compounds on seeds and seedings of lettuce. Table S4. Allelopathic activities of compounds on seeds and seedings of wheat.
