# Supplementary material for: Allelopathy and Identification of Volatile Components from the Roots and Aerial Parts of Astragalus mongholicus Bunge
Source: Plants (Basel). 2024 Jan 20;13(2):317. doi: 10.3390/plants13020317 (PMC10819805; doi:10.3390/plants13020317)
Supplement: Supplementary file 1 [file plants-13-00317-s001.zip › plants-2817776-supplementary.pdf]

Table S1 Identity of volatile compounds in *Astragalus mongholicus* (*AM*)

Classes	NO.	R.T. (minutes)	Name	CAS	RI[a]	RI[b]	Relative content%	
							AMA	AMR
Alcohols	1	2.88	Methyl Alcohol	67-56-1	931	903	2.62	/
	2	3.45	Ethanol	64-17-5	951	932	0.2	0.44
	3	8.11	(*E*)-2-Pentenal	1576-87-0	1115	1127	0.41	/
	4	9.48	1-Penten-3-ol	616-25-1	1159	1158	2.34	1.19
	5	12.07	1-Pentanol	71-41-0	1244	1250	0.2	0.87
	6	13.75	1-Pentanol, 2-methyl-	105-30-6	1300	1293	0.21	/
	7	14.03	(*Z*)-2-Penten-1-ol	1576-95-0	1310	1318	0.21	/
	8	14.21	(*Z*)-2-Hexen-1-ol, acetate	56922-75-9	1316	1315	0.46	/
	9	14.99	1-Hexanol	111-27-3	1344	1355	0.32	8.23
	10	15.79	(*Z*)-3-Hexen-1-ol	928-96-1	1373	1382	0.46	/
	11	16.04	3-Octanol	589-98-0	1382	1393	0.81	/
	12	16.38	(*E*)-2-Hexen-1-ol	928-95-0	1394	1406	1.2	1.07
	13	16.76	2-Octanol	123-96-6	1411	1411	/	6.79
	14	17.63	1-Octen-3-ol	3391-86-4	1438	1450	0.4	4.52
	15	19.91	Linalool	78-70-6	1533	1547	0.79	/
	16	20.29	1-Octanol	111-87-5	1549	1557	0.49	/
	17	21.63	(*E*)-2-Octen-1-ol	18409-17-1	1606	1613	0.72	/
	18	22.12	1-Nonen-4-ol	35192-73-5	1629	/	/	0.65
	19	22.13	4-Methyl-5-decanol	213547-15-0	1630	/	0.97	/
	20	25.79	2,6-Octadien-1-ol, (*Z*)-3,7-dimethyl-	106-25-2	1797	1797	0.23	/
	21	26.79	Geraniol	106-24-1	1846	1847	1.79	/
	22	27.26	Benzyl alcohol	100-51-6	1869	1870	2.86	0.53
	23	28.03	Phenylethyl Alcohol	60-12-8	1906	1907	1.88	0.92
Lipids	1	9.87	Hexanoic acid, methyl ester	106-70-7	1172	1184		1.46
	2	12.45	Acetic acid, hexyl ester	142-92-7	1256	1273	0.77	/
	3	13.77	(*Z*)-3-Hexen-1-ol, acetate	3681-71-8	1291	1316	5.59	0.65
	4	13.93	n-Caproic acid vinyl ester	3050-69-9	1306	1312	0.25	0.55
	5	15.51	1-Octen-3-yl-acetate	2442-10-6	1363	1379	0.94	/
	6	16.47	Butanoic acid, hexyl ester	2639-63-6	1397	1414	0.38	/
	7	17.67	(*Z*)-Butanoic acid, 3-hexenyl ester	16491-36-4	1443	1455	2.24	/
	8	17.99	cis-3-Hexenyl-methylbutyrate	53398-85-9	1456	1492	1	/
	9	18.03	(*E*)-Butanoic acid, 2-hexenyl ester	53398-83-7	1457	1463	0.93	/
	10	21.48	Benzoic acid, methyl ester	93-58-3	1599	1612		0.33
	11	22.45	(*Z*)-Hexanoic acid, 3-hexenyl ester	31501-11-8	1645	1646	0.58	/
	12	22.77	(*E*)-Hexanoic acid, 2-hexenyl	53398-86-0	1660	1660	0.15	/

			ester					
	13	23.97	Acetic acid, phenylmethyl ester	140-11-4	1716	1720	0.73	/
	14	24.68	Geranyl acetate	105-87-3	1748	1752	0.4	/
	15	24.94	Methyl salicylate	119-36-8	1759	1765	1.26	0.63
	16	25.88	Acetic acid, 2-phenylethyl ester	103-45-7	1802	1813	0.66	/
	17	27.28	2,2,4-Trimethyl-1,3-pentane diol diisobutyrate	6846-50-0	1869	/	0.2	0.35
	18	39.12	(3S,3aR)-3-Butyl-3a,4,5,6-tetrahydroisobenzofuran-1(3H)-one	4567-33-3	2496	/	0.19	/
	19	40.27	Senkyunolide	63038-10-8	2545	/	0.23	/
**Aldehydes**	1	4.19	Pentanal	110-62-3	977	979	0.29	0.23
	2	6.9	Hexanal	66-25-1	1074	1083	7.05	5.68
	3	9.73	Heptanal	111-71-7	1167	1185	0.21	/
	4	10.21	2-Butenal, 3-methyl-	107-86-8	1183	1214	/	0.17
	5	10.83	(*E*)-2-Hexenal	6728-26-3	1185	1216	1.36	2.39
	6	13.85	(*Z*)-2-Heptenal	57266-86-1	1303	1322	0.22	1.26
	7	15.96	(*E,E*)-2,4-Hexadienal	142-83-6	1375	1404	0.28	0.69
	8	16.32	5-Ethylcyclopent-1-enecarboxaldehyde	36431-60-4	1392	1410	0.2	0.29
	9	16.73	(*E*)-2-Octenal	2548-87-0	1407	1429	0.34	1.23
	10	17.57	Furfural	98-01-1	1439	1461	0.37	0.44
	11	19.05	Benzaldehyde	100-52-7	1497	1520	6.24	5.33
	12	19.45	(*E*)-2-Nonenal	18829-56-6	1513	1534	/	0.31
	13	21.49	1-Cyclohexene-1-carboxaldehyde, 2,6,6-trimethyl-	432-25-7	1600	1611	0.61	/
	14	22.92	(*Z*)-2,6-Octadienal, 3,7-dimethyl-	106-26-3	1667	1680	0.59	0.2
	15	23.36	(*E,E*)-2,4-Nonadienal	5910-87-2	1687	1699	0.38	0.72
	16	24.08	2,6-Octadienal, (*E*)-3,7-dimethyl-	141-27-5	1721	1732	0.22	0.83
**Phenolic**	1	33.32	2-Methoxy-4-vinylphenol	7786-61-0	2182	2188	0.44	0.22
	2	38.86	Z-Butylidenephthalide	72917-31-8	2482	/	0.24	0.18
**Acids**	1	17.26	Acetic acid	64-19-7	1427	1449	2.05	0.54
	2	19.68	Propanoic acid	79-09-4	1523	1535	/	0.15
	3	24.45	Pentanoic acid	109-52-4	1737	1733	/	0.25
	4	26.57	Hexanoic acid	142-62-1	1835	1846	/	6.78
	5	29.14	2-Hexenoic acid	1191-04-4	1962	1980	/	0.22
	6	31.06	Octanoic acid	124-07-2	2061	2060	0.18	0.27
	7	38.52	Iminodiacetic acid	142-73-4	2463	/	1.42	/
**Ketones**	1	5.14	1-Penten-3-one	1629-58-9	1011	1019	3.66	1.6
	2	11.87	3-Octanone	106-68-3	1237	1253	1.03	0.28

	3	12.78	2-Octanone	111-13-7	1267	1287	/	0.41
	4	13.23	1-Octen-3-one	4312-99-6	1282	1301	2.28	0.95
	5	16.17	3-Octen-2-one	1669-44-9	1386	1411	0.2	0.6
	6	20.3	3,5-Octadien-2-one	38284-27-4	1549	1522	0.4	/
	7	22.11	Acetophenone	98-86-2	1629	1647	0.18	0.15
	8	22.44	6,7-Dodecanedione	13757-90-9	1644	/	/	0.80
	9	28.49	3-Buten-2-one, 4-(2,6,6-trimethyl-1-cyclohe xen-1-yl)-	14901-07-6	1929	1967	2.32	0.2
	10	29.52	3-Buten-2-one, 4-(2,2,6-trimethyl-7-oxabicy clo[4.1.0]hept-1-yl)-	23267-57-4	1981	1962	1.47	/
	11	34.66	1H-Pyrrole-2,5-dione, 3-ethyl-4-methyl-	20189-42-8	2254	2244	0.27	/
**Alkenes**	1	11.82	Styrene	100-42-5	1235	126 1	/	0.17
	2	13.35	(*E*)-4,8-Dimethylnona-1,3,7-t riene	19945-61-0	1286	1311	0.21	/
	3	22.77	Benzene, 1-ethenyl-4-methoxy-	637-69-4	1660	1680	/	0.3
**Heterocycles**	1	3.7	Furan, 2-ethyl-	3208-16-0	960	951	1.93	1.63
	2	11.1	Furan, 2-pentyl-	3777-69-3	1212	1232	0.82	2.94
	3	13.2	cis-2-(2-Pentenyl)furan	70424-13-4	1281	1273	0.17	/
	4	16.9	Pyrazine, 2-methoxy-3-(1-methylethyl)-	25773-40-4	1413	1427	0.64	0.21
	5	18.72	Pyrazine, 2-methoxy-3-(1-methylprop yl)-	24168-70-5	1484	1502	/	1.8
	6	19.3	Pyrazine, 2-methoxy-3-(2-methylprop yl)-	24683-00-9	1507	1518	0.62	/
	7	19.53	Furan, 2-butyltetrahydro-	1004-29-1	1517	/	/	0.88
**Others**	1	5.59	Toluene	108-88-3	1027	1042	/	0.17
	2	16.71	Benzene, 1,3-bis(1,1-dimethylethyl)-	1014-60-4	1406	1427	0.28	/

Notes: R.T, retention time; RC, relative content; AMA, aerial parts of *A. mongholicus*; AMR, roots of *A. mongholicus*; RI, Retention Index; a, calculated RI; b, RI in NIST or literatures.

Table S2. Allelopathic activities of compounds on seeds and seedings of *AM*

Compounds	Treatment (mg/L)	Seeds		Seedings		SI
		GR (%)	GP	RL（mm）	SL（mm）	
1-Hexanol	0	85.55 ± 4.01a	73.33 ± 5.09a	8.47 ± 2.37a	6.78 ± 0.35ab	0.00
	50	63.33 ± 1.67b	60.00 ± 2.89a	9.56 ± 0.74a	6.19 ± 0.25b	-0.10
	100	83.33 ± 4.41ab	68.33 ± 1.67a	8.38 ± 0.97a	6.45 ± 0.22b	-0.04
	200	81.67 ± 8.33ab	68.33 ± 4.41a	11.37 ± 1.58a	6.77 ± 0.38ab	0.06
	400	61.67 ± 1.67b	31.67 ± 6.01b	9.01 ± 1.21a	8.47 ± 0.58a	-0.13
(*E*)-2-hexenal	0	85.55 ± 4.01a	73.33 ± 5.09a	8.47 ± 2.37ab	6.78 ± 0.35a	0.00
	50	73.33 ± 14.81a	63.33 ± 9.28a	8.57 ± 0.90ab	6.63 ± 0.27a	-0.07
	100	70.00 ± 2.89a	53.33 ± 6.67ab	11.07 ± 1.00a	6.93 ± 0.31a	-0.03
	200	78.33 ± 6.01a	61.67 ± 4.41a	6.76 ± 0.61bc	6.49 ± 0.32a	-0.12
	400	60.00 ± 7.64a	25.00 ± 5.00b	3.82 ± 0.31c	6.07 ± 0.29a	-0.40
(*E*,*E*)-2,4-Decadienal	0	85.55 ± 4.01a	73.33 ± 5.09a	8.47 ± 2.37a	6.78 ± 0.35ab	0.00
	50	81.67 ± 4.41a	68.33 ± 12.02a	10.30 ± 0.90ab	7.66 ± 0.26a	0.06
	100	56.67 ± 10.93ab	50.00 ± 10.41ab	4.77 ± 0.74d	6.53 ± 0.48ab	-0.28
	200	71.67 ± 8.82ab	33.33 ± 13.64ab	5.70 ± 0.65cd	5.54 ± 0.38b	-0.30
	400	43.33 ± 4.41b	15.00 ± 2.89b	7.67 ± 0.51bc	5.94 ± 0.38b	-0.38
Hexanal	0	85.55 ± 4.01a	73.33 ± 5.09a	8.47 ± 2.37a	6.78 ± 0.35a	0.00
	50	75.00 ± 7.64a	68.33 ± 9.28a	10.84 ± 1.24a	6.05 ± 0.21ab	0.00
	100	85.00 ± 2.89a	66.67 ± 10.14a	10.12 ± 1.80a	5.50 ± 0.37ab	-0.02
	200	80.00 ± 5.00a	63.33 ± 7.26a	5.82 ± 0.85a	4.29 ± 0.67b	-0.22
	400	90.00 ± 2.89a	80.00 ± 2.89a	7.88 ± 1.08a	6.10 ± 0.43ab	-0.08
Eugenol	0	85.55 ± 4.01a	73.33 ± 5.09a	8.47 ± 2.37b	6.78 ± 0.35a	0.00
	50	71.67 ± 3.33a	61.67 ± 6.01a	11.74 ± 1.46a	6.97 ± 0.23a	0.02
	100	70.00 ± 5.00a	70.00 ± 5.00a	7.13 ± 1.27b	7.38 ± 0.38a	-0.07
	200	71.67 ± 1.67a	66.67 ± 1.67a	7.04 ± 0.69b	7.13 ± 0.14a	-0.09
	400	71.67 ± 6.67a	53.33 ± 17.40a	3.43 ± 0.32c	6.58 ± 0.33a	-0.26

Notes: GR, germination rate; GP, germination potential; RL, radicle length; SL, shoot length; SI, synthetical allelopathic index; Different lowercase letters indicate significant difference at $p < 0.05$ level.

Table S3. Allelopathic activities of compounds on seeds and seedings of lettuce

Compounds	Treatment (mg/L)	Seeds		Seedings		SI
		GR (%)	GP	RL（mm）	SL（mm）	
1-Hexanol	0	94.45 ± 2.22a	77.78 ± 4.01a	13.04 ± 1.07b	5.81 ± 0.35d	0.00
	50	95.00 ± 2.89a	55.00 ± 7.64ab	20.61 ± 0.83a	7.95 ± 0.25bc	0.17
	100	96.67 ± 1.67a	58.33 ± 11.67ab	19.59 ± 1.16a	8.91 ± 0.37abc	0.20
	200	93.33 ± 1.67ab	50.00 ± 10.41ab	20.70 ± 1.27a	9.23 ± 0.27ab	0.20
	400	80.00 ± 5.77b	31.67 ± 6.67b	20.36 ± 1.05a	9.88 ± 0.47a	0.13
(*E*)-2-Hexenal	0	94.45 ± 2.22a	77.78 ± 4.01a	13.04 ± 1.07a	5.81 ± 0.35d	0.00
	50	93.33 ± 1.67a	63.33 ± 4.41ab	17.95 ± 0.89a	7.72 ± 0.25bc	0.13
	100	90.00 ± 2.89a	61.67 ± 6.01ab	17.21 ± 0.97a	7.89 ± 0.21bc	0.10
	200	83.33 ± 7.26a	38.33 ± 11.67b	17.51 ± 1.45a	8.65 ± 0.32b	0.05
	400	0.00 ± 0.00b	0.00 ± 0.00c	16.82 ± 1.30a	10.46 ± 0.35a	-0.23
(*E*,*E*)-2,4-Deca dienal	0	94.45 ± 2.22a	77.78 ± 4.01a	13.04 ± 1.07a	5.81 ± 0.35bc	0.00
	50	90.00 ± 2.89ab	53.33 ± 6.01ab	14.48 ± 1.27a	6.98 ± 0.26ab	-0.01
	100	88.33 ± 4.41ab	25.00 ± 2.89bc	11.37 ± 1.08a	7.06 ± 0.18a	-0.01
	200	76.67 ± 4.41b	0.00 ± 0.00c	4.02 ± 0.37b	5.12 ± 0.34c	-0.34
	400	0.00 ± 0.00c	0.00 ± 0.00c	4.98 ± 0.61b	6.83 ± 0.32ab	-0.40
Hexanal	0	94.45 ± 2.22a	77.78 ± 4.01a	13.04 ± 1.07a	5.81 ± 0.35c	0.00
	50	96.67 ± 1.67a	70.00 ± 5.00a	15.90 ± 0.71a	7.39 ± 0.18bc	0.11
	100	95.00 ± 2.89a	73.33 ± 1.67a	14.20 ± 0.72a	7.64 ± 0.28b	0.09
	200	98.33 ± 1.67a	73.33 ± 3.33a	14.58 ± 0.97a	9.72 ± 0.62a	0.19
	400	95.00 ± 2.89a	58.33 ± 1.67a	15.05 ± 0.56a	11.27 ± 0.58a	0.21
Eugenol	0	94.45 ± 2.22a	77.78 ± 4.01a	13.04 ± 1.07b	5.81 ± 0.35bc	0.00
	50	88.33 ± 4.41a	56.67 ± 13.33ab	22.80 ± 1.56a	6.68 ± 0.25ab	0.14
	100	90.00 ± 0.00a	40.00 ± 2.89b	19.71 ± 1.37a	6.31 ± 0.26abc	0.01
	200	86.67 ± 1.67a	41.67 ± 1.67b	20.51 ± 1.00a	6.14 ± 0.32abc	0.02
	400	91.67 ± 4.41a	45.00 ± 0.00b	3.12 ± 0.33c	5.20 ± 0.30c	-0.31

Notes: GR, germination rate; GP, germination potential; RL, radicle length; SL, shoot length; SI, synthetical allelopathic index; Different lowercase letters indicate significant difference at $p < 0.05$ level..

Table S4. Allelopathic activities of compounds on seeds and seedings of wheat

Compounds	Treatment (mg/L)	Seeds		Seedings		SI
		GR (%)	GP	RL（mm）	SL（mm）	
1-Hexanol	0	96.67 ± 1.67a	90.00 ± 5.00a	20.95 ± 2.81a	23.46 ± 1.46a	0.00
	50	95.00 ± 2.89a	81.67 ± 4.41a	3.89 ± 0.51b	23.63 ± 3.26a	-0.23
	100	95.00 ± 2.89a	90.00 ± 2.89a	3.02 ± 0.44b	10.52 ± 3.23bc	-0.36
	200	88.33 ± 4.41a	81.67 ± 10.93a	2.36 ± 0.38b	6.73 ± 2.64bc	-0.45
	400	81.67 ± 7.26a	61.67 ± 8.82a	2.58 ± 0.25b	2.66 ± 1.92c	-0.56
(*E*)-2-Hexenal	0	96.67 ± 1.67a	90.00 ± 5.00a	20.95 ± 2.81bc	23.46 ± 1.46bc	0.00
	50	93.33 ± 1.67ab	76.67 ± 7.26a	19.26 ± 2.81bc	28.24 ± 2.40bc	-0.01
	100	98.33 ± 1.67a	91.67 ± 1.67a	14.75 ± 2.75bc	31.44 ± 2.85ab	0.02
	200	86.67 ± 4.41ab	73.33 ± 8.82a	12.96 ± 1.63bc	24.06 ± 2.66bc	-0.16
	400	80.00 ± 5.77b	75.00 ± 5.00a	9.93 ± 2.53c	18.65 ± 1.79c	-0.27
(*E*,*E*)-2,4-Decadienal	0	96.67 ± 1.67a	90.00 ± 5.00a	20.95 ± 2.81a	23.46 ± 1.46a	0.00
	50	93.33 ± 4.41a	90.00 ± 5.00a	10.68 ± 2.01ab	27.44 ± 3.58a	-0.09
	100	93.33 ± 3.33a	88.33 ± 1.67a	17.38 ± 3.87ab	27.27 ± 4.48a	-0.01
	200	95.00 ± 0.00a	95.00 ± 0.00a	9.52 ± 1.90b	27.97 ± 2.02a	-0.08
	400	98.33 ± 1.67a	98.33 ± 1.67a	9.08 ± 1.55b	18.79 ± 3.50a	-0.16
Hexanal	0	96.67 ± 1.67a	90.00 ± 5.00a	20.95 ± 2.81ab	23.46 ± 1.46abc	0.00
	50	91.67 ± 4.41a	88.33 ± 6.01a	15.81 ± 3.64abc	27.01 ± 2.46ab	-0.04
	100	90.00 ± 2.89a	88.33 ± 1.67a	3.62 ± 0.35d	21.31 ± 2.31abc	-0.25
	200	90.00 ± 2.89a	80.00 ± 5.77a	5.10 ± 0.75cd	16.73 ± 3.32bc	-0.31
	400	85.00 ± 2.89a	78.33 ± 4.41a	5.51 ± 1.12cd	13.50 ± 3.44c	-0.35
Eugenol	0	96.67 ± 1.67ab	90.00 ± 5.00a	20.95 ± 2.81b	23.46 ± 1.46cd	0.00
	50	96.67 ± 3.33ab	90.00 ± 5.77a	18.24 ± 3.19b	26.12 ± 2.88bc	0.00
	100	96.67 ± 1.67ab	93.33 ± 4.41a	10.31 ± 1.84bc	23.81 ± 2.45cd	-0.11
	200	98.33 ± 1.67a	95.00 ± 0.00a	4.09 ± 0.63c	23.34 ± 2.06cd	-0.18
	400	93.33 ± 1.67ab	81.67 ± 6.67a	2.75 ± 0.22c	14.48 ± 3.40d	-0.35

Notes: GR, germination rate; GP, germination potential; RL, radicle length; SL, shoot length; SI, synthetical allelopathic index; Different lowercase letters indicate significant difference at $p < 0.05$ level.
